# An enrichment-based approach to interpreting metabolomic data using differential metabolomic profiles within the iDMET framework

**DOI:** 10.1186/s12859-026-06456-6

**Published:** 2026-04-27

**Authors:** Rira Matsuta, Hiroyuki Yamamoto, Atsushi Fukushima, Sho Tabata, Hideki Makinoshima, Tomoyoshi Soga, Rintaro Saito, Eisuke Hayakawa

**Affiliations:** 1 Institute for Advanced Biosciences, Keio University, Tsuruoka, Yamagata 997-0052 Japan; 2 Systems Biology Program, Graduate School of Media and Governance, Keio University, Fujisawa, Kanagawa 252-8520 Japan; 3 Human Metabolome Technologies, Inc., Tsuruoka, Yamagata 997-0052 Japan; 4 Graduate School of Life and Environmental Sciences, Kyoto Prefectural University, Kyoto, Kyoto 606-8522 Japan; 5 RIKEN Information R&D and Strategy Headquarters, Wako, Saitama 351-0198 Japan; 6 Tsuruoka Metabolomics Laboratory, National Cancer Center, Tsuruoka, Yamagata 997-0052 Japan; 7 Shonai Regional Industry Promotion Center, Tsuruoka, Yamagata 997-0052 Japan; 8 Division of Translational Research, Exploratory Oncology Research, and Clinical Trial Center, National Cancer Center, Kashiwa, Chiba 277-8577 Japan; 9 Human Biology-Microbiome-Quantum Research Center, Keio University, Tsuruoka, Yamagata 997-0052 Japan; 10 Department of Bioscience and Bioinformatics, Faculty of Computer Science and Systems Engineering, Kyushu Institute of Technology, Iizuka, Fukuoka 820-8502 Japan; 11 RIKEN Center for Sustainable Resource Science, Yokohama, Kanagawa 230-0045 Japan

**Keywords:** Metabolomics, Enrichment analysis, Differential metabolomic profile, Reanalysis, Cancer

## Abstract

**Background:**

Pathway enrichment analysis is a crucial method for the biological interpretation of metabolomic data by identifying associations between altered metabolites and biological pathways. However, such traditional approaches often rely on a limited set of predefined metabolic pathways, resulting in a low likelihood of discovering pathways associated with a given metabolic profile. To overcome this limitation, we extended our previously developed iDMET methodology to incorporate a broader range of metabolite sets, including those derived from differential metabolomic profiles. This enhanced approach, termed iDMET+, significantly expands dataset diversity and size, increasing the likelihood of discovering associated metabolite sets for a given metabolic profile, thereby enables more biological insights to be obtained from the metabolic profile.

**Results:**

We validated iDMET+ through case studies on three diseases: clear cell renal cell carcinoma, colorectal cancer, and small cell lung cancer. First, using a clear cell renal cell carcinoma study as input, iDMET+ correctly identified another study of the same disease that involved metabolomic analysis. This pair of studies was identified as relevant in our previous iDMET results, showing the consistency between iDMET+ and iDMET. Second, using the metabolomic profile of colorectal cancer as input, iDMET+ identified not only another metabolomic study of the same cancer but, surprisingly, also metabolomic studies on prostate cancer and a high-fat diet. These studies focused on MYC-driven metabolic reprogramming, which was also a major focus of the input study. In both case studies, related studies were enriched because the differential metabolomic profiles of directly associated studies were part of the metabolite set. In contrast, the small cell lung cancer study highlighted limitations in dataset coverage—the absence of directly relevant differential metabolomic profiles resulted in fewer enriched metabolite sets. Nevertheless, the analysis of commonly altered metabolites still yielded some meaningful results. Metabolite alterations associated with inhibition of the purine salvage pathway were observed, suggesting potential involvement in tumor metabolic reprogramming.

**Conclusions:**

These results demonstrate that iDMET+ offers broader biologically relevant information than the conventional pathway-based approaches and has the potential to uncover biologically significant findings by searching across diverse datasets. This work also identifies areas of improvements for iDMET+.

**Supplementary Information:**

The online version contains supplementary material available at 10.1186/s12859-026-06456-6.

## Background

Pathway enrichment analysis, which is also referred to as functional enrichment analysis, is a critical method for evaluating the statistical significance of overlaps between a set of molecules exhibiting remarkable changes in expression in a specific studied condition (e.g., a disease) and those already known to be involved in specific metabolic pathways or functions. Such analysis supports biological interpretation of the observed changes. This method is widely used in the fields of genomics, transcriptomics, and proteomics, with representative methods including Gene Set Enrichment Analysis (GSEA) [1] and Over-Representation Analysis (ORA) [2].

Compared with the development of enrichment analyses in these omics fields, the equivalent development in the field of metabolomics is still in its infancy. However, enrichment analysis based on ORA is a widely used method to elucidate the enrichment of metabolites of interest in specific metabolic pathways. Meanwhile, MetaboAnalyst [3] is a well-known platform that implements various enrichment analysis methods, including ORA, and enables researchers to rapidly analyze large-scale metabolomic data to derive biological insights. For instance, in the work of Ramsay et al. analyzing salivary metabolomics in head and neck cancer patients undergoing radiotherapy, enrichment analysis revealed that five metabolic pathways—including pyruvate metabolism, glycolysis, and several amino acid-related pathways—were significantly altered by the treatment [4].

Enrichment analysis has thus become indispensable for interpreting metabolomics data and is expected to play an increasingly significant role in future research. However, this approach is still markedly limited by the fact that metabolite sets for performing such analysis are not comprehensively organized, and most of the sets are focused only on well-known metabolic pathways. This is in stark contrast to the situation in genomics, transcriptomics, and proteomics, where genes are organized into gene ontology functional categories, and large numbers of gene sets that are regulated in specific conditions are accumulated in databases such as MSigDB [5].

To integrate heterogeneous metabolome datasets by connecting differential analyses across studies, we previously proposed iDMET [6], a novel computational method that systematically finds pairs of metabolomic studies between which there is overlap in the metabolites showing remarkable changes in their levels, thus revealing novel relationships between these studies. In fact, iDMET discovered an interesting relationship between a pair of studies, one analyzing change in metabolomic profile after treatment with the drug geatolisib for breast cancer and the other analyzing that by the drug M_4_N for Hodgkin lymphoma. Given that both drugs are associated with the PI3K/AKT/mTOR signaling pathway, iDMET was suggested to be effective for discovering novel pairs of studies that are biologically related to each other. During the work on iDMET, we collected metabolomic profiles from 27 studies and created sets of metabolites exhibiting increases and decreases in the conditions given in each study. Here, each set was analogous to curated gene sets employed in enrichment analyses in other omics research, specifically corresponding to the chemical and genetic perturbations category in the curated gene set collection of MSigDB [5].

The objective of this study was to develop iDMET+ , which involved using iDMET to enhance the applicability of ORA-based enrichment analysis by ultimately incorporating a broader range of metabolite sets. These sets corresponded not only to specific metabolic pathways, but also to those revealed by data-driven approaches to reflect subsets of pathways, combinations of multiple pathways, or sets of functionally related metabolites, making ORA-based enrichment analysis more practical in metabolomics. Specifically, we focused on cancer metabolomics on a range of different cancers. We conducted systematic collection and curation of metabolites described in research papers, classifying them based on increases and decreases according to specific criteria, and added them to those collected in our previous pilot study on iDMET [6]. We also incorporated the metabolite sets that showed correlated changes in a pair of studies, which were found during analyses by iDMET. The size of the metabolite sets prepared in this study increased up to approximately five times larger than those used in the original study in which iDMET was established (Table 1). This dataset formed the basis for our analysis. The original version of iDMET was intended to search for pairs of metabolomic studies related to each other among the collected studies in our database. In contrast, iDMET+ was designed to accept query metabolites from a study prepared by the user and searches against other relevant studies in the database, making the system useful to a broad range of researchers studying metabolomics.

**Table 1:** Comparison of the number of datasets and studies used for iDMET and iDMET+

Data resource	iDMET	iDMET+
PubMed	25 studies	58 studies
	(39 datasets)	(94 datasets)
MetaboLights	2 studies	15 studies
		(16 datasets)
Metabolomics Workbench	–	52 studies
		(57 datasets)

**Fig. 1:**
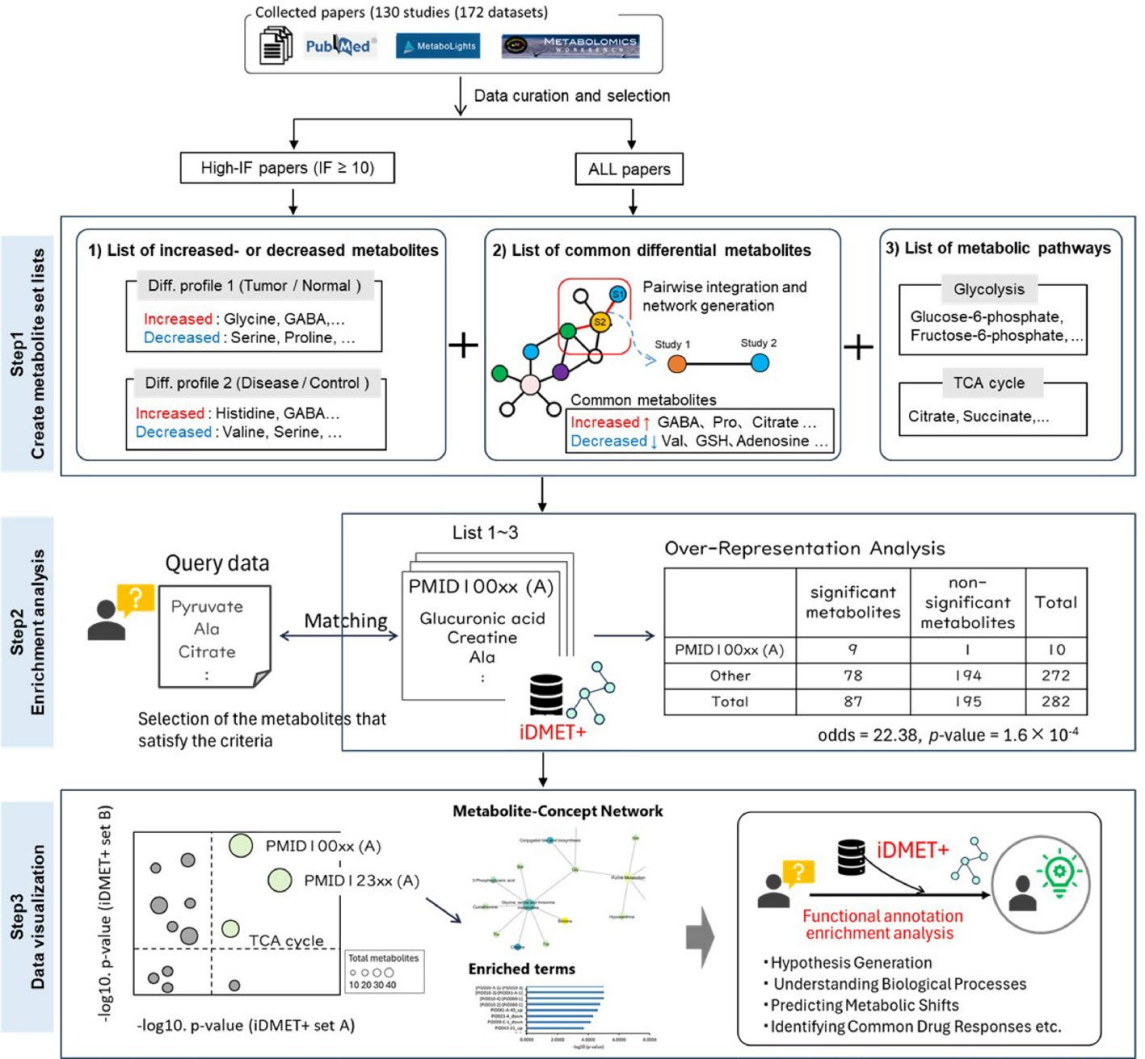
Overview of the iDMET+ workflow: data integration via iDMET and its application to enrichment analysis. An overview of the iDMET+ workflow. The procedure in iDMET+ consists of three main steps. In Step 1, metabolomic datasets are collected from published studies and public repositories. Metabolite sets are then constructed based on the differential metabolomic profiles in list 1, the common differential metabolomic profiles in list 2, and the existing metabolic pathways in list 3. The differential metabolomic profile in list 1 was generated from studies published in journals with an Impact Factor of ≥ 10. The common differential metabolomic profiles in list 2 were generated by identifying metabolites shared across differential metabolomic profiles through pairwise comparisons of all collected studies. This procedure was based on the iDMET method for inter-study comparison, which identifies metabolites consistently observed across pairwise study comparisons, thereby providing more reliable differential metabolomic profiles. The pathway-based metabolite set in list 3 was curated based on KEGG pathway information. In Step 2, ORA-based enrichment analysis was performed on user-submitted differential metabolomic profiles as a query, using the iDMET+ metabolite set constructed in Step 1 by integrating list 1, list 2, and list 3. In Step 3, results of the enrichment analysis or networks of selected metabolites are visually represented to facilitate biological interpretation. The Metabolite-Concept Network illustrates the relationships between metabolites and enriched metabolite sets, providing an intuitive view of their associations. The iDMET+ workflow is described in detail in the Materials and Methods section

We collected cancer metabolome data from public databases and selected a total of 27 studies for iDMET in the previous work and 130 studies for iDMET+ in our present work. These 130 studies were used to generate the metabolite set lists for enrichment analysis. Details of the list generation procedure are provided in the Methods section and Fig. 1

To demonstrate the utility of iDMET+ , we applied the framework to three case studies including different cancer types [7–9]. In each case, iDMET+ identified potentially relevant studies and provided insights that aligned with and further extended previous findings or experimental backgrounds. These results validate the ability of iDMET+ to extract meaningful connections from only differential metabolomic profiles without prior information about study metadata, such as disease types or study designs.

## Materials and methods

### Summary of analytical workflow

The workflow of the enrichment analysis using iDMET+ applied in this study is presented in Fig. 1. This workflow consists of three steps, which are described in detail below.

### Step 1: consolidation of metabolite sets

Step 1 of the workflow (Fig. 1) involves the construction of three types of metabolite sets: list 1 (differential metabolomic profiles within individual studies), list 2 (common differential metabolomic profiles across studies), and list 3 (metabolic pathway–based metabolite sets). To generate these lists, we first systematically collected cancer metabolomics data following the approach of Matsuta et al. [6]. Data were sourced from PubMed, MetaboLights [10], and Metabolomics Workbench [11] using keyword searches, followed by manual screening of the identified study entries (top of Fig. 1). Specifically, we included cancer metabolomics studies using analytical platforms such as gas chromatography–mass spectrometry (GC–MS) and liquid chromatography–mass spectrometry (LC–MS), while excluding lipidomics studies [6]. This process identified 130 studies (58 from PubMed and 72 from repositories) with metabolomic data available (Supplementary Table 1).

To ensure the quality and reliability of the collected studies, those published in journals with an Impact Factor of 10 or higher were used to construct list 1. This resulted in a final set of 30 studies (24 from PubMed and 6 from repositories) (Table 2) [7, 8, 12–39]. For studies involving more than three conditions for metabolomic measurements, differential metabolomic profiles were created from all possible pairwise combinations of the conditions. When studies reported significant metabolite differences between conditions, these results were directly adopted. For each resulting differential metabolomic profile, the participating metabolites were categorized into those that increased and those that decreased, generating two metabolite sets from each differential profile. In total, we obtained 570 metabolite sets (list 1).

**Table 2:** List of 30 cancer metabolomics studies used for generating differential metabolomic profiles

	PMID	Journal	Metabolite detection method	Total number of metabolites targeted in this study (remaining rate)	References
1	26766592	Cancer Cell	GC–MS, LC–MS/MS	489 (84.5%)	[7]
2	28,847,964	Proc Natl Acad Sci USA	CE-TOFMS, LC–MS/MS	127–186 (70.7–77.9%)	[8]
3	31554818	Nat Commun	UPLC-MS/MS	127 (44.3%)	[12]
4	31171880	Nat Med	CE-TOFMS	255 (56.4%)	[13]
5	29490947	Cancer Res	CE-TOFMS	89–104 (78.6–100%)	[14]
6	29533781	Cancer Cell	CE-TOFMS, LC–MS	6–51 (50.0–75.0%)	[15]
7	30482722	EBioMedicine	1H-NMR	52 (85.2%)	[16]
8	20861191	Cancer Res	NMR	15 (71.4%)	[17]
9	31953253	Gut	CE-TOFMS	278 (52.9%)	[18]
10	27133130	Cell Metab	CE-TOFMS	94 (92.2%)	[19]
11	27345838	Cell Stem Cell	CE-TOFMS	91 (82.0%)	[20]
12	27345495	Nat Commun	CE-TOFMS	273 (53.0%)	[21]
13	29084919	Clin Cancer Res	CE-TOFMS	74 (76.3%)	[22]
14	19458066	Cancer Res	CE-TOFMS	91 (80.5%)	[23]
15	26980435	J Exp Clin Cancer Res	CE-TOFMS	58–66 (69.6–75.0%)	[24]
16	36450243	Cell Rep	CE-TOFMS	87 (83.7%)	[25]
17	31577958	Cell Rep	CE-TOFMS	10–94 (38.5–71.8%)	[26]
18	28514652	Cell Rep	CE-TOFMS	174 (73.4%)	[27]
19	23643539	Cell Rep	CE-TOFMS	93–123 (70.3–75.6%)	[28]
20	28122247	Cell Rep	CE-TOFMS	98 (92.5%)	[29]
21	25855294	Nature	CE-TOMS, LC–MS	34–102 (42.0–91.1%)	[30]
22	30903027	Nat Commun	LC–MS	33 (75.0%)	[31]
23	30996345	Nature	LC–MS/MS	18–154 (50.7–85.7%)	[32]
24	31281534	Theranostics	GC–MS	45 (19.7%)	[33]
25	22628425	Cancer Res	GC–MS, LC–MS/MS	221–243 (82.2–90.3%)	[34]
26	33990571	Nat Commun	LC–MS	101–117 (71.1–88.0%)	[35]
27	28082280	Clin Cancer Res	NMR	20 (95.2%)	[36]
28	23236214	Clin Cancer Res	GC–MS	49 (94.2%)	[37]
29	33060170	Cancer Res	LC–MS/MS	18 (100%)	[38]
30	32726636	Cell Rep	LC–MS	61 (81.3%)	[39]

These studies were selected based on journal quality (Impact Factor ≥ 10) and used for constructing differential metabolomic profiles, as described in Fig. 1. Detection methods included gas chromatography (GC), liquid chromatography (LC), mass spectrometry (MS), time-of-flight mass spectrometry (TOFMS), nuclear magnetic resonance (NMR), and capillary electrophoresis (CE). Literature searches were performed in February 2023. Numbers (%) in parentheses represent the rates of metabolites that remained after matching all reported metabolites with the curated list of metabolite names and synonyms

Next, we generated a set of common differential metabolomic profiles, referred to as list 2, based on the iDMET approach [6] to capture metabolites consistently altered across studies (see *Supplementary Methods* for details). Specifically, we performed pairwise comparisons of all collected differential metabolomic profiles to identify metabolites that were shared between multiple studies, thereby generating more reliable differential metabolomic profiles. From the pairs of common differential metabolomic profiles generated by the iDMET approach, we selected those that were more amenable to biological interpretation (see *Supplementary Methods* for details). This selection aimed to identify pairs that were not only statistically significant but also biologically meaningful to some extent. As a result, a final set of 25 common differential metabolomic profiles was obtained. Based on these profiles, list 2, consisting of metabolite sets grounded in the results of iDMET analysis, was created. For existing metabolic pathways (list 3), we created in-house 36 metabolite sets based on representative metabolic pathways in KEGG [40, 41].

### Step 2: enrichment analysis

In Step 2, shown in the middle section of Fig. 1, we integrated three types of metabolite sets (i.e., metabolites with significant changes in differential profiles generated from individual studies; list 1), those shared by pairs of differential profiles from different studies according to iDMET analysis (list 2), and those in known metabolic pathways (list 3). Our metabolite sets were used as a basis to perform enrichment analysis using ORA in iDMET+ . The significance of enrichment was assessed using Fisher’s exact test, and *q*-values were calculated using the Benjamini–Hochberg procedure. In particular, iDMET+ matches the list of increased and decreased metabolites prepared by a user (query data), together with a background list, to our metabolite sets (list 1–3). In conventional pathway enrichment analyses, increased and decreased metabolites are not always treated separately. In contrast, iDMET+ incorporates the direction of change into each metabolite set. Therefore, enrichment analyses were performed separately for increased and decreased metabolites to ensure directional consistency between the query data and the metabolite sets. The background list was defined as all metabolites detected in the user-provided query dataset, rather than all theoretically measurable metabolites, in order to account for differences in analytical platforms and detection coverage across studies. The result of ORA is returned to the user, which includes metabolite sets enriched in the query metabolite list and the statistical significance of such enrichments along with their visualization (see Step 3).

To demonstrate the effectiveness of the enrichment analysis by iDMET+ , we conducted case studies using three query datasets. The first case study utilized one of the differential profiles used in our previous iDMET study, namely the comparison of cancerous and normal tissues in clear cell renal cell carcinoma (ccRCC) [7] to show how the result of iDMET can be reproduced in iDMET+ . The second case study examined normal and cancerous tissues in colorectal cancer (CRC) patients [8], while the third case study used metabolomic data comparing tumor tissues xenografted from HPRT1-KO cells and WT cells in small cell lung cancer (SCLC) [9]. In each case study, query metabolites (i.e., differential metabolites) were selected based on two-group comparisons. ORA-based enrichment analysis in iDMET+ was then performed separately for increases and decreases metabolites in the query, using only list1 in the ccRCC case study and list 1–3 in the CRC and SCLC case studies. All metabolite sets used in this study are freely available in the repository (https://github.com/riramatsuta/iDMET_enrich). Users can download the iDMET+ R scripts from the repository and execute them in R to perform enrichment analyses using their own query datasets.

### Step 3: visualization of enrichment analysis

In iDMET+ , a match between the query set and a given metabolite set is assumed to be biologically relevant only if differential metabolomic profiles in the metabolite set are enriched. To find such matches, the list of query metabolites was divided into those that were increased and those that were decreased, and enrichment analysis was conducted separately for each. To facilitate an integrated understanding of both groups, we created a scatter plot where the x-axis represents the log_10_(*p*-values) from the enrichment analysis for the increased metabolites, and the y-axis represents the log_10_(*p*-values) for the decreased metabolites, as shown in Step 3 of Fig. 1. This visualization helps the user to identify studies with enrichment in both increased and decreased metabolites. Additionally, to facilitate interpretation of the enrichment results, we visualized the associations between significantly enriched metabolite sets and metabolites in the query dataset using a network graph. In this network graph, nodes represent enriched metabolite sets and individual metabolites, and edges indicate relationships between metabolite sets and their constituent metabolites. Metabolite nodes are colored according to the direction of fold change (increased or decreased). Scatter plots and network graphs were generated with the R packages igraph [42] and clusterProfiler [43]. These tools provided clear and intuitive representations, enhancing the interpretability of the analysis outcomes.

## Results and discussion

### Case study 1: comparison of cancerous and normal tissues in clear cell renal cell carcinoma (ccRCC)

First, we compared how iDMET+ and our previously developed iDMET associate the metabolite set derived from the differential metabolomic profile from Hakimi’s work on ccRCC [7] with other metabolomic studies or metabolite sets. iDMET searched for statistically significant matches of increased and decreased metabolites for all pairs of the metabolite sets in the iDMET database. Each significance calculation was based on both increased and decreased metabolites in the pair. As a result, one of the significant pair had a metabolite set from Hakimi’s work on ccRCC. Meanwhile, iDMET+ performed enrichment analyses based on ORA using Hakimi’s metabolomic profile as a query to collected metabolite sets in the iDMET+ database. In this analysis, we only used a metabolite set derived from differential metabolic profiles (list 1). Metabolites in a query and each metabolite set in the database were classified into those that were increased and decreased in each given condition, prior to conducting enrichment analyses in iDMET+ . Thus, the statistical significances of the matches to each metabolite set were calculated separately for groups of increased and decreased metabolites in the query.

**Fig. 2:**
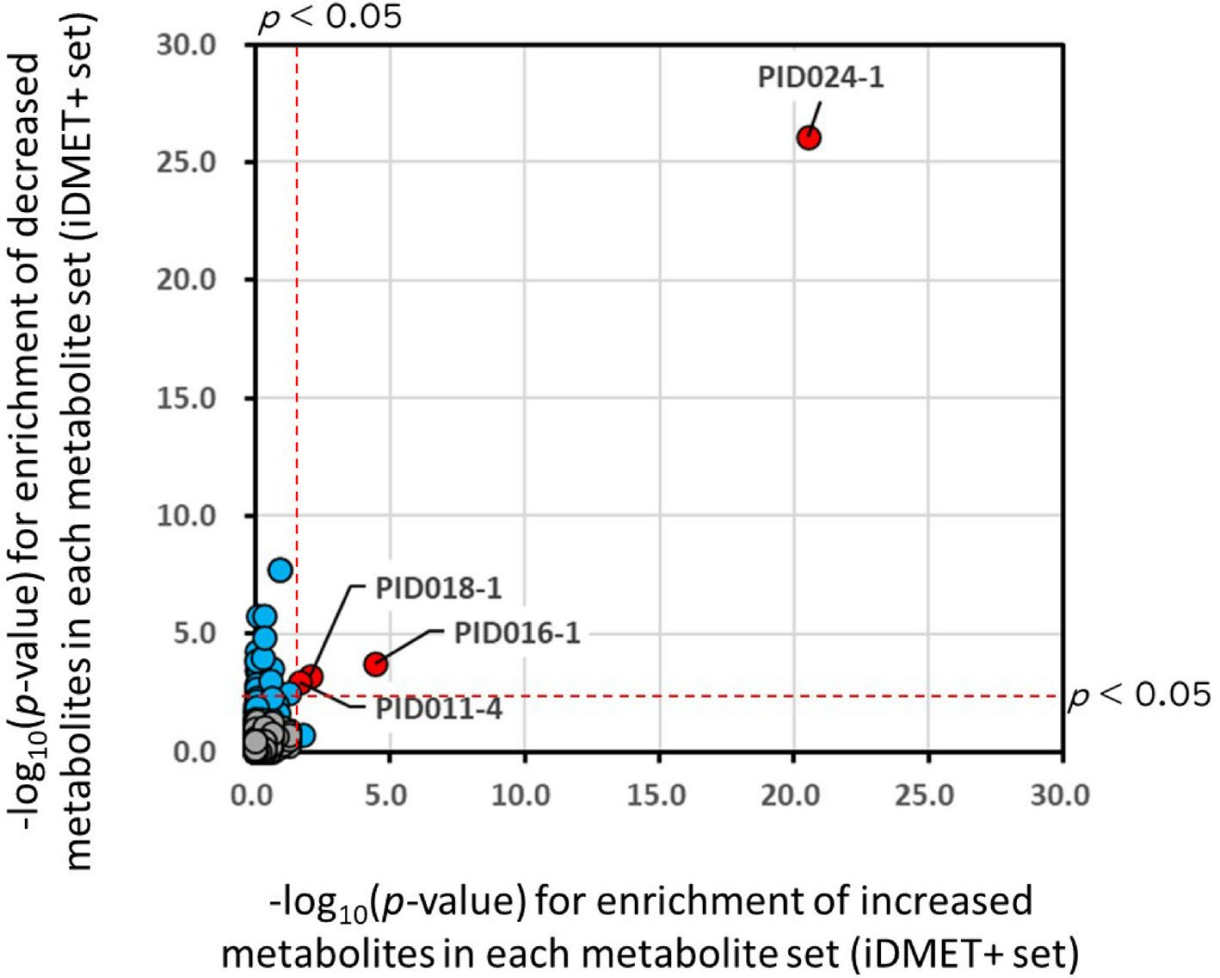
Visualization of the results of enrichment analysis implemented in iDMET+ using Hakimi’s ccRCC dataset as a query. Results of enrichment analysis using Hakimi’s ccRCC dataset [7] as a query. Each circle represents an individual metabolite set in the iDMET+ database. The enrichment of each metabolite set in the increased and decreased metabolites in the query is statistically evaluated separately. The x- and y-axes represent the −log_10_(*p*-values) from ORA for increased and decreased metabolites in the query, respectively. Blue plots represent metabolite sets that were significantly enriched (*p* < 0.05) in only one of the conditions, while red plots represent metabolite sets that were significantly enriched in both conditions. The most significantly enriched metabolite set (PID024-1) [44] corresponds to the same study used in our original iDMET analysis [6]

The differential metabolomic profile comparing cancerous and normal tissues in ccRCC contained 501 regulated metabolites, among which 246 were classified as increased metabolites (ratio > 1.2) in the cancerous tissues and 167 were classified as decreased ones (ratio < 1/1.2). These were used as two groups of metabolites in the query in ORA-based enrichment analysis of iDMET+ . For each metabolite set in the target iDMET+ database, we checked whether the member metabolites overlapped with both the increased and decreased groups of metabolites in the query. If not, the metabolite set was excluded, resulting in 424 sets used as the target of the query in the subsequent analyses (Fig. 2).

The ORA-based enrichment analysis in iDMET+ revealed that 5 out of 424 metabolite sets were significantly enriched (*p* < 0.05) for the increased metabolites in the query, while 76 metabolite sets showed significant enrichment for the decreased metabolites. By taking the overlap of these two sets of results, we identified four metabolite sets that were significantly enriched in both increased and decreased metabolites in the query (*p* < 0.05): PID024-1 [44], PID018-1 [45], PID016-1 [46], and PID011-4 [47].

Among these metabolite sets, PID024-1 exhibited the most notable results. For the increased and decreased metabolites, the *p*-values of enrichment analysis were 2.93 × 10^−21^ and 8.28 × 10^−27^, respectively. The corresponding *q*-values were 1.24 × 10^−18^, 3.51 × 10^−24^ and the odds ratios were 9.91 and 12.07. The number of matched metabolites was 102 out of 119 for the increased metabolites and 86 out of 113 for the decreased ones. PID024-1 is based on a cohort study comparing metabolomic data from human ccRCC and normal kidney tissues using GC–MS and LC–MS [44]. In our previous iDMET analysis, the similarity between PID024-1 and Hakimi’s ccRCC data—the dataset used as the query in this study—was the highest among all pairwise comparisons. This supports the consistency between the finding of the present study and our previous work and demonstrates the effectiveness of iDMET+ as well as our previous iDMET approach in identifying biologically meaningful differential metabolomic profiles.

The other three differential metabolomic profiles (PID018-1, PID016-1, and PID011-4) were also identified in our previous iDMET network analysis as being significantly associated with Hakimi’s ccRCC data, further confirming the consistency of these findings.

### Case study 2: MYC-related metabolic changes in colorectal cancer (CRC)

**Fig. 3:**
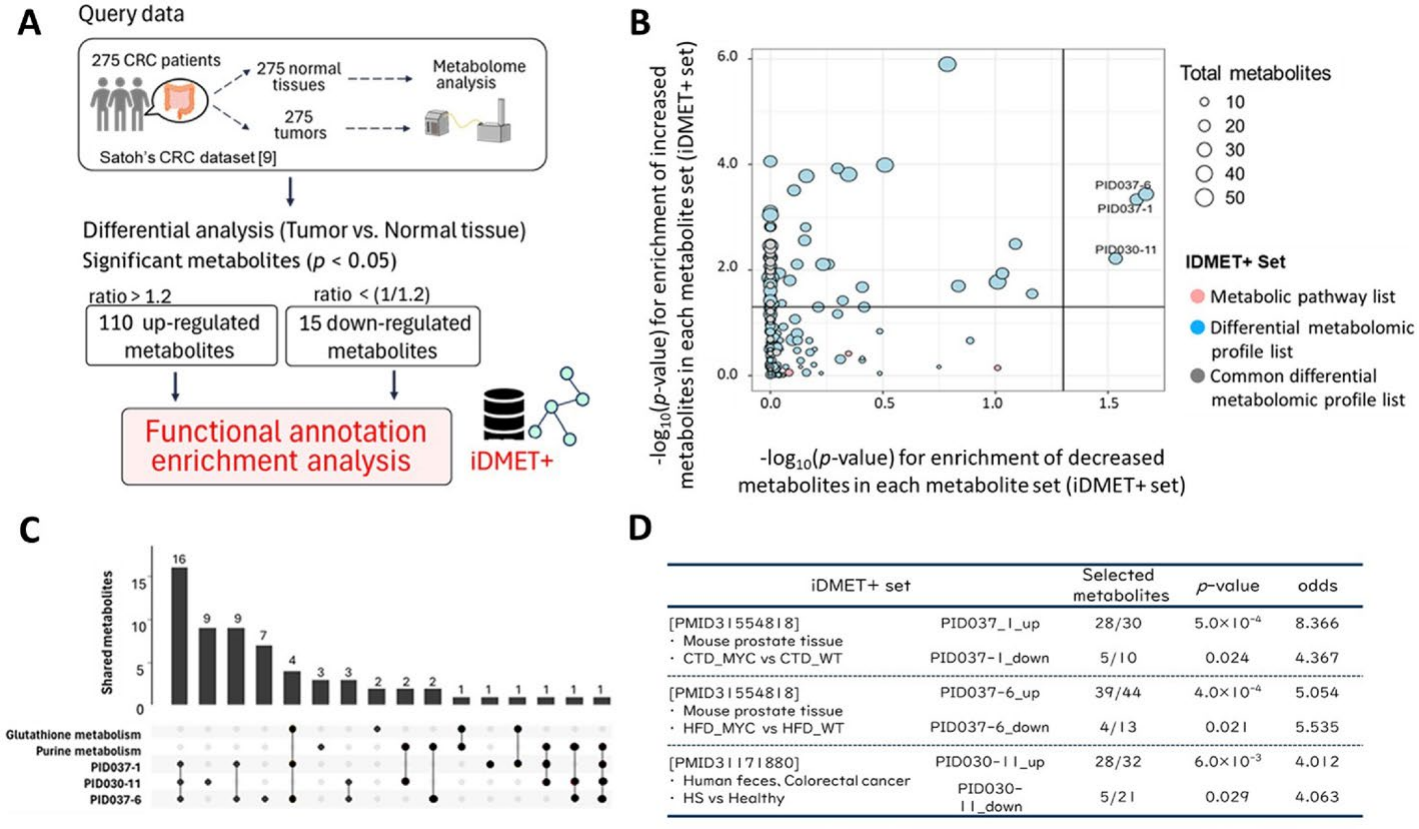
Analysis of Satoh’s CRC dataset using the iDMET+ workflow. Satoh’s CRC dataset [8] was analyzed using the iDMET+ workflow. **A** A schematic overview of the iDMET+ workflow in Satoh’s CRC dataset. **B** Scatter plot of −log_10_(*p*-values) from ORA-based enrichment analysis for increased (y-axis) and decreased (x-axis) metabolites. Analyses were performed separately for the increased and decreased metabolite sets. Each dot represents a metabolite set derived from a differential metabolomic profile in the iDMET+ database. **C** UpSet plot showing the overlap of metabolites across significantly enriched metabolite sets. The black bars indicate the number of metabolites shared among the profiles as indicated by the black dots below the x-axis. A single dot indicates metabolites unique to one profile, while connected dots represent metabolites shared across multiple profiles. **D** Table summarizing the results of the enrichment analysis. ORA was performed separately for increased and decreased metabolites, and the table lists metabolite sets that were significantly enriched under both conditions. The column “Selected metabolites” indicates the number of metabolites from each set that were identified in the enrichment analysis

In the second case study, we performed enrichment analyses based on ORA implemented in iDMET+ , using metabolomic data reported by Satoh et al. [8] as a query (Fig. 3A). Satoh’s CRC metabolome data were acquired from both cancerous and normal tissues of 275 patients. Metabolome analysis was performed using CE-TOFMS, which detected a total of 161 metabolites. Among them, 110 metabolites were significantly increased (ratio > 1.2 and *p* < 0.05) in cancerous tissues, while 15 metabolites were significantly decreased (ratio < 1/1.2 and *p* < 0.05) (Fig. 3A). ORA-based enrichment analysis in iDMET+ was performed separately for the increased and decreased metabolites in the query, using three types of metabolite sets (lists 1–3). Among the metabolic pathways (list 3), only 3 out of 35 were significantly enriched for the increased metabolites (*p* < 0.05): Glutathione metabolism (*p* = 0.044, *q* = 0.201), Glucogenic amino acids (*p* = 0.010, *q* = 0.096), and Essential amino acids (*p* = 0.029, *q* = 0.165). For the decreased metabolites, no metabolic pathways were significantly enriched. Enrichment analysis performed using both increased and decreased metabolites did not identify any significantly enriched metabolic pathways (*p* < 0.05). These limited findings highlight the inherent limitations of pathway-based enrichment analysis, which relies on a relatively small and predefined set of metabolites. In addition, as demonstrated in this case study, when a relatively large number of metabolites show statistically significant changes, the proportion of significant metabolites within pathways becomes similar to that outside pathways. This reduces the likelihood that metabolites in specific pathways are enriched, resulting in fewer significantly enriched metabolite sets.

Compared with pathway-based enrichment analysis, the use of differential metabolomic profiles from metabolite set lists 1 and 2 revealed a broader range of biologically relevant information. Within metabolite set list 1, which was derived from differential metabolomic profiles, the metabolites in 93 out of 520 sets were significantly enriched (*p* < 0.05) in the increased metabolites in the query, all with odds ratios greater than 4 (Fig. 3B). For the decreased metabolites, only three metabolite sets derived from differential metabolomic profiles were significantly enriched. Among these significantly enriched metabolite sets, three differential metabolomic profiles (PID037-1, PID037-6, and PID030-11) were significantly enriched for both increased and decreased metabolites in the query (Fig. 3C, D). Analysis using metabolite set list 2, which was based on common differential metabolomic profiles derived from our previous iDMET approach, identified 17 out of 50 metabolite sets as significantly enriched (*p* < 0.05) for the increased metabolites, while none was enriched for the decreased metabolites (Fig. 3B).

In our analysis using metabolite set list 1, the metabolites in PID030-11 were significantly enriched in both increased and decreased metabolites in the query, with *p*-values of 0.006 and 0.029, *q*-values of 0.082 and 1.00, and the odds ratios of 4.012 and 4.063, respectively (Fig. 3D). The metabolite set PID030-11 was derived from a study comparing metabolomic profiles of CRC patients to healthy controls using CE-TOFMS [13]. The number of matched metabolites between the metabolite set and the metabolites in the query was 28 out of 32 for the increased metabolites and 5 out of 21 for the decreased metabolites. Because PID030-11 is based on a study of CRC and was statistically significantly enriched for both increased and decreased metabolites, this result suggests that our method can capture cancer-specific metabolic changes. This illustrates that biologically meaningful differential metabolomic profiles can be identified using only fluctuations in metabolite levels, without relying on additional metadata such as experimental conditions or patient background.

Furthermore, high odds ratio associations were observed for PID037-1 and PID037-6, which are derived from studies of different cancer types (Fig. 3B, D). Both differential metabolomic profiles are derived from studies that investigated a high-fat diet and prostate cancer progression using UPLC-MS/MS [12], and both represent models with MYC overexpression. PID037-1 compares metabolomic profiles of MYC-overexpressing mice with WT controls, while PID037-6 compares those of high-fat-diet-fed MYC-overexpressing mice with those of wild-type controls.

For PID037-1, 28 out of 30 increased metabolites matched with the significantly increased metabolites in the query from Satoh’s CRC dataset (*p* = 0.0005, *q* = 0.239, odds ratio = 8.36), while the corresponding proportion for the decreased ones was 5 out of 10 (*p* = 0.023, *q* = 1.00, odds ratio = 4.36) (Fig. 3B, D). Similarly, for PID037-6, 39 out of 44 metabolites matched with the increased metabolites in the query (*p* = 0.0004, *q* = 0.021, odds ratio = 5.05) (Fig. 3B, D). These results suggest that the observed metabolic changes are primarily attributable to MYC overexpression rather than dietary effects.

Importantly, the query dataset originated from a CRC study focusing on MYC, but this information was not used in the analysis. Nevertheless, PID037-1 and PID037-6, from different cancer types and related to MYC-driven metabolic reprogramming, showed high odds ratio associations (Fig. 3D). This demonstrates that iDMET+ can detect biologically relevant metabolic changes directly from differential metabolomic profiles and identify MYC-driven alterations in CRC, providing results consistent with existing knowledge (Fig. 3).

### Case study 3. Metabolome data from mouse models of small cell lung cancer (SCLC)

**Fig. 4:**
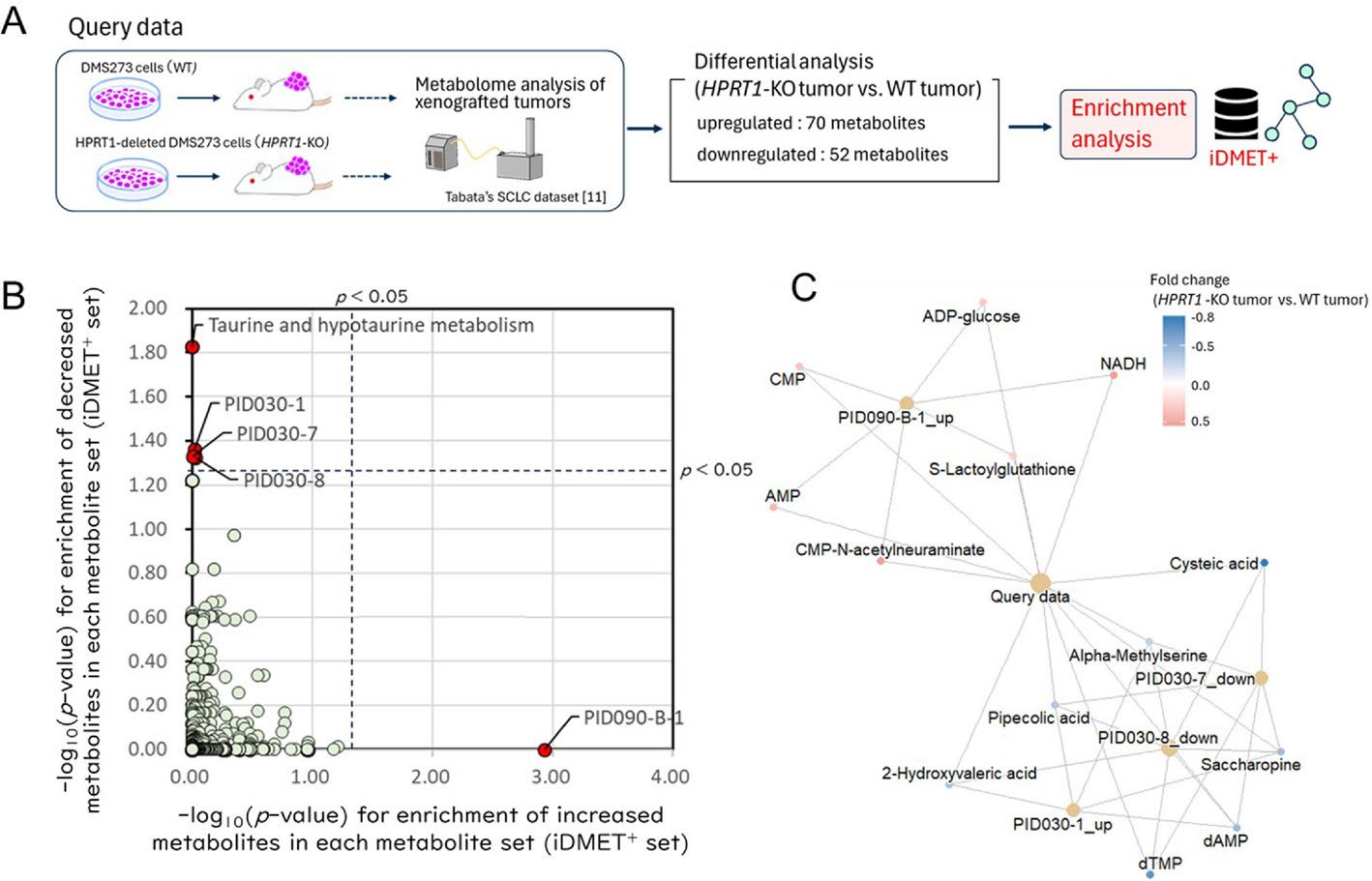
Analysis of Tabata’s SCLC dataset using the iDMET+ workflow. Tabata’s SCLC dataset [9] was analyzed using the iDMET+ workflow. **A** Overview of the analytical workflow. **B** Scatter plots showing −log_10_(*p*-values) from ORA-based enrichment analysis for increased (x-axis) and decreased (y-axis) metabolites. Each dot represents a metabolite set derived from the differential metabolomic profile in the iDMET+ database. **C** Network visualization using cnetplot from the R package clusterProfiler. The network showing associations between significantly enriched metabolite sets derived from differential metabolomic profiles (large nodes) and the metabolites in the query associated with the metabolite sets (small nodes). Metabolite nodes are colored according to fold changes, with red and blue intensities indicating increased and decreased levels of metabolites, respectively

In the third case study, ORA-based enrichment analysis implemented in iDMET+ was performed for Tabata’s SCLC data [9] (Fig. 4A). The dataset focused on the metabolic profiles of purine nucleotide biosynthesis in SCLC, comparing tumor tissues derived from HPRT1-KO and WT cells. A total of 210 metabolites were detected, among which 33 metabolites were significantly increased (*p* < 0.05) and 17 were significantly decreased (*p* < 0.05), both of which were used as a query to iDMET+ . Among the increased metabolites in the query, the metabolite set in only one metabolic pathway was significantly enriched (*p* < 0.05), and no metabolite sets derived from differential metabolomic profiles were significantly enriched. Among the decreased metabolites, no metabolite set in the metabolic pathway or those derived from differential metabolomic profiles were significantly enriched. Enrichment analysis performed using both increased and decreased metabolites did not identify any significantly enriched metabolic pathways (*p* < 0.05).

Unlike in case studies 1 and 2, there were no common metabolite sets from the metabolomic differential profiles that were enriched in both the increased and decreased metabolites in case study 3. One likely reason for this is a contextual mismatch between the query metabolites and the predefined list of metabolite sets used in iDMET+. While the query—Tabata’s SCLC data—was derived from xenografted tumors, the predefined list of metabolite sets included only two sets that were derived from SCLC-related studies: those of Makinoshima et al. [14] and Morita et al. [15]. Both were based on in vitro cell line models and focused on specific metabolic contexts, such as PI3K inhibitor sensitivity or PKM1-driven reprogramming. Differences in experimental conditions, biological contexts, and pathway coverage may have limited the detection of statistically significant enrichment of the metabolite sets in the query.

None of the metabolite sets derived from differential metabolomic profiles was significantly enriched in the initial analysis. Therefore, as in our previous iDMET study [6], metabolites were selected solely based on the mean ratio between groups, without considering *p*-values, and the analysis was performed again. In the analysis using metabolic pathways, no metabolite set in metabolic pathways was found to be enriched in the increased metabolites in the query (*p* < 0.05), whereas 1 out of 35 metabolite sets in metabolic pathways was significantly enriched in the decreased metabolites (Fig. 4B). For differential metabolomic profiles, 1 out of 520 metabolite sets from differential metabolomic profiles was significantly enriched (*p* < 0.05) in the increased metabolites, and 3 out of 520 were significantly enriched in the decreased metabolites. The significantly enriched differential metabolomic profiles for increased metabolites did not overlap with those significantly enriched for decreased metabolites. The metabolite sets derived from the common differential metabolomic profiles generated from iDMET results were significantly enriched (*p* < 0.05) in neither the increased nor the decreased metabolites (Fig. 4B).

As shown in Fig. 4C, some metabolites were shared across multiple studies from which differential metabolomic profiles were derived. Among the metabolites found to be decreased in multiple studies, dAMP, dTMP, and saccharopine are associated with purine/pyrimidine metabolism and mitochondrial amino acid metabolism. These pathways depend on purine salvage mediated by HPRT1, and metabolic changes similar to those caused by HPRT1 deficiency have also been observed in the early stages of cancer. This suggests that reduced HPRT1 activity may contribute to metabolic reprogramming in early tumorigenesis. Furthermore, decreases in deoxyribonucleotide metabolites such as dAMP and dTMP were observed, while increases in ribonucleotide metabolites including AMP and CMP were also noted. These changes may reflect the accumulation of precursors due to suppressed DNA synthesis, and an imbalance in nucleotide metabolism caused by inhibition of the purine salvage pathway. A study by Townsend et al. reported the overexpression of HPRT1 in colorectal cancer samples compared with the level in normal tissue [48].

Variations were observed in non-nucleotide metabolites, including S-lactoylglutathione, cysteic acid, saccharopine, and 2-hydroxyvaleric acid. Saccharopine and 2-hydroxyvaleric acid have been reported to be associated with the gut microbiota [49]. S-Lactoylglutathione and cysteic acid are associated with intracellular oxidative stress and antioxidant responses [50, 51]. These findings suggest that the inhibition of HPRT1 may affect not only the purine salvage pathway but also the dynamics and utilization of microbiota-derived metabolites in the host, as well as metabolism related to oxidative stress. Such changes may be an important aspect of cancer-associated metabolic reprogramming. Although the direct relationship between HPRT1, SCLC, and the gut microbiota remains largely unclear and requires further investigation, information from altered metabolites shared by multiple studies may still provide various biological insights even when no differential metabolomic profiles with positive correlations with both increased and decreased metabolites in the query are found.

In case studies 1 and 2, we confirmed that, when the same metabolite set derived from the same differential metabolomic profile was significantly enriched in both increased and decreased metabolites in the query, it facilitated a more direct biological interpretation (Figs. 2, 3). This result was consistent with our previous findings using the iDMET approach, in which biologically meaningful interpretations were possible when two differential metabolomic profiles shared both increased and decreased metabolites.

In contrast, in case study 3, no metabolite set derived from differential metabolomic profiles was enriched in both the increased and decreased metabolites in the query (Fig. 4B). One of the reasons for this was the absence of directly relevant metabolite sets in our iDMET+ database. This suggests that further expansion of the metabolite sets is necessary. Still, metabolite sets from differential metabolomic profiles were separately enriched in increased and decreased metabolites, which enabled us to derive biologically meaningful interpretations when considering them together (Fig. 4B, C). This highlights the strength of our approach in enabling biological interpretation, even when directly relevant differential metabolomic profiles are limited.

### Limitations and future directions

iDMET+ provides a framework for enrichment analysis using differential metabolomic profiles, yet some limitations remain. First, it relies solely on ORA, a widely used method in metabolomics. Because ORA ignores the magnitude of change for each metabolite, it may miss subtle but important patterns. Future updates will incorporate methods like GSEA [1] to enhance sensitivity. Second, study-derived metabolite sets are not always easy to use. Many sets lack clear labels, often forcing users to re-read the original papers for biological context. To simplify this, we plan to develop an automated system that adds clear descriptions to every set. Third, the data selection process may involve some degree of subjective judgment. Currently, the selection of differential metabolomic profiles depends on curator judgment, which may affect database consistency. Future refinements could include the development of an automated system and objective criteria for profile selection, thereby improving database transparency and reducing potential bias. Finally, the current database is focused on cancer-related metabolic research, which may limit the ability to detect patterns in other metabolic states. Expanding data collection to include a broader range of diseases and conditions could make iDMET+ a more versatile platform for the metabolomics community.

## Conclusion

This paper presents iDMET+ , an extended approach to ORA-based enrichment analysis by incorporating both conventional metabolic pathways and metabolite sets derived from differential metabolomic profiles. In this work, the iDMET+ framework was validated through three case studies. In the ccRCC case, the results of iDMET+ were consistent with our previous iDMET study. The CRC case showed that the method could detect MYC-driven metabolic changes and provided clear biological interpretations that were straightforward and consistent with prior knowledge. Finally, in the SCLC case, although no remarkable enrichment of metabolite sets was found—likely owing to limited relevance of the dataset—metabolites shared across separate analyses still provided a basis for biological interpretation. These findings demonstrate that iDMET+ provides a practical and interpretable framework for enrichment analysis in metabolomics, offering a complementary perspective to traditional metabolic pathway-based analyses.

## Supplementary Information

Below is the link to the electronic supplementary material.

Supplementary Material 1: Metabolomic datasets were collected from PubMed, MetaboLights [10], and Metabolomics Workbench [11] through keyword searches, followed by manual screening (for details, see Fig. 1 and Materials and Methods). Numbers (%) in parentheses indicate the proportion of metabolites that remained after matching all reported metabolites in each study with the curated list of metabolite names and synonyms prepared for this work.

Supplementary Material 2: We calculated the similarity of each pair of differential metabolome profiles, and we selected pairs having remarkable odds ratios and p-values (p < 0.05, odds ratio > 4). We set the following thresholds: ratio > 1.2 (upper threshold) or ratio < 1/1.2 (lower threshold).

Supplementary Material 3

## Data Availability

Metabolomic data used in this study were obtained from MetaboLights, Metabolomics Workbench, and the supplementary materials of published articles cited in Supplementary Methods [1–33, 58–83]. The corresponding accession numbers and PubMed IDs for all datasets are listed in Supplementary Table 1. The processed datasets used for the analyses in this study are publicly available at https://github.com/riramatsuta/iDMET_enrich. The main metabolite set lists used in this study, including `iDMET_pathwaylist.csv`, are also provided in this repository. These files can be freely accessed and used for enrichment analyses in metabolomic studies. iDMET+ is provided as R scripts, which users can download and run in R using their own query datasets.
