# Supplementary material for: An enrichment-based approach to interpreting metabolomic data using differential metabolomic profiles within the iDMET framework
Source: BMC Bioinformatics. 2026 Apr 27;27:131. doi: 10.1186/s12859-026-06456-6 (PMC13267349; doi:10.1186/s12859-026-06456-6)
Supplement: Supplementary file 3 — Supplementary Material 3 [file 12859_2026_6456_MOESM3_ESM.docx]

**Supplementary Methods**

In this study, we grouped together metabolite sets that had differential metabolomic profiles in common using the iDMET approach. The overall workflow is shown in Supplementary Figure 1, the details of which are described below.

Using all 130 studies, we first converted metabolome data reported in each study into differential metabolomic profiles [1-85]. Then, pairs of differential metabolomics profiles with increased or decreased metabolites in common were extracted based on the following criteria: ratios of metabolite levels in both differential profiles are above the threshold of 1.2 (i.e., commonly increased metabolites) or below the threshold of 1/1.2 (i.e., commonly decreased metabolites). We also performed the same process with thresholds of 1.5 and 1/1.5, respectively.

From the 1,111 differential metabolomic profiles generated through the above iDMET analysis, we selected pairs of differential metabolomic profiles where two profiles in each pair came from different studies and had at least five increased and five decreased metabolites in common. The selected pairs were further filtered based on the significance of the number of differential metabolites in common using the following criteria: *p*-value < 0.05 (chi-square test) and odds ratio > 4. The 1,428 pairs meeting these criteria were manually curated, and comparisons involving similar conditions (e.g., Control 1 vs. Control 2, Treatment 1 vs. Treatment 2) in each differential profile were excluded. Pairs with interpretable biological significance were retained, resulting in a final set of 25 differential metabolomic profiles [12], [13], [17], [18], [21], [24], [31], [47], [58], [59], [61], [65], [69], [71], [72], [76], [78], [79], [80], [83]. Based on these profiles, metabolite sets grounded in the results of iDMET analysis were created, dividing the metabolites into increased and decreased sets.

Next, we briefly describe the 25 differential metabolomic profiles used for biological interpretation. The comparisons PID017-1 [17] and P024-1 [24] both represent differential metabolomic profiles between tumor and normal tissues in clear cell renal cell carcinoma (ccRCC). Notably, the log2 odds ratio for this comparison was as high as 11.97 (*p*-value = 6.20×10^−38^), indicating a striking contrast at the metabolite level. Although derived from independent studies, the two comparisons involve the same cancer type, and the high concordance in observed metabolic alterations highlights the reproducibility and biological relevance of the findings.

As with the comparison between PID017-1 [17] and PID024-1 [24], several significant positive associations were observed between studies targeting the same cancer type (Table S2), including comparisons between PID010-4 [13] and PID080-1 [58], PID010-2 [13] and PID080-1 [58], PID010-3 [13] and PID080-1 [58], PID010-2 [13] and PID081-A-45 [59], and PID010-4 [13] and PID106-D-2. Among these, the pair with the highest log2 odds ratio was the comparison between PID010-4 [13] and PID080-1 [58] (log2 odds ratio = 9.20, *p*-value = 9.80 × 10⁻⁹). In addition, significant associations were identified between datasets involving the same cancer type (lung cancer) but different subtypes (Table S2), such as PID081-C-1 [59] vs. PID088-1 [61], PID010-4 [4] vs. PID081-A-45 [59], PID080-1 [58] vs. PID081-A-45 [59], and PID010-3 [4] vs. PID081-A-1 [59].

The comparison between PID031-1 [31] and PID081-C-1 [59] is also notable. Both datasets represent metabolomic profiles comparing tumor and normal tissues. PID031-1 compares post-gastrectomy patients with healthy controls in gastric cancer, while PID081-C-1 compares tumor and normal tissues in small cell lung cancer (SCLC). Although the cancer types differ, several metabolites showed consistent increases or decreases in tumor tissues relative to the levels in normal tissues in both cases. These findings suggest that certain metabolic features may be commonly altered across different types of cancer.

The comparisons between PID107-10 [80] and PID105-E-1 [78], as well as PID107-19 [80] and PID105-E-1, are based on studies investigating the impact of drug resistance on metabolic alterations in cancer cell lines. PID107-10 involves malignant pleural mesothelioma cells, in which upregulation of thymidylate synthase has been associated with pemetrexed resistance, and acetylation of H3K27 has been implicated in this process. In PID105-E-1, suppression of glutamine synthetase in ovarian cancer cells is linked to cisplatin resistance, and epigenomic changes are suggested to induce resistance via metabolic reprogramming. Notably, acetylation of H3K27 has also been reported to regulate the expression of cancer-related genes and contribute to tumor progression in breast cancer cells [86]. These findings collectively suggest that common epigenetic modifications may lead to shared metabolic alterations across different cancer types and drug resistance mechanisms.

The comparison between PID096-A-5 [69] and PID103-5 [76] involves differential metabolomic profiles between tumor and normal tissues in colorectal cancer and lung cancer, respectively. Although these studies focus on different cancer types, both demonstrate that several metabolites show consistent increases or decreases in tumor tissues compared with the levels in normal tissues, suggesting shared metabolic alterations across cancer types.

The comparison between PID021-1 [21] and PID110-1 [83] involves differential metabolomic profiles between tumor and normal tissues related to liver cancer, both measured using CE-TOFMS. PID021-1 compares hepatocellular carcinoma with normal tissue, while PID110-1 compares intrahepatic cholangiocarcinoma with surrounding non-tumor tissue. The log2 odds ratio for the comparison was 5.009 (*p*-value = 3.0 × 10⁻⁵). Metabolites commonly elevated in tumor tissues across both datasets included 1-methylnicotinamide, L-arginine, hypotaurine, kynurenine, L-methionine, and N6-acetyllysine. Conversely, commonly decreased metabolites included betaine, carnitine, choline, nicotinamide, AMP, ATP, and NAD⁺. These results suggest consistent metabolic alterations between tumor and non-tumor tissues, potentially reflecting shared metabolic features characteristic of liver cancer.

The comparison between PID017-1 [17] and PID018-3 [18] yielded a negative association, with a log2 odds ratio of −4.32 (*p*-value = 0.003). Both metabolomic analyses were conducted using GC-MS and LC-MS. PID017-1 represents the metabolomic profile comparing tumor and normal tissues in ccRCC. PID018-3 represents the metabolomic profile of L428 cells (a human Hodgkin lymphoma cell line) treated with MN4 (tetra-O-methyl nordihydroguaiaretic acid). Several metabolites that increased following MN4 treatment overlapped with those elevated in normal tissues in PID017-1. This suggests that MN4 treatment may shift the metabolic state of L428 cells toward a more normal profile.

PID018-3 [18] also showed a negative association with both PID006-A-1 [12] and PID006-B-1 [12], with a log2 odds ratio of −4.29 (*p*-value = 0.002) in each case. The PID006 study investigated differential metabolomic profiles between renal cancer and normal tissues using multiple types of biospecimens: PID006-A-1 represents serum samples, while PID006-B-1 represents tissue samples. Metabolites increased by MN4 treatment overlapped with those elevated in normal samples (compared with the levels in cancer) in both serum and tissue datasets from PID006. These findings suggest that differential metabolomic profiles can capture cancer-specific metabolic alterations consistently across different sample types, highlighting their potential utility for understanding disease-associated metabolic changes.

Significant positive associations were observed in the comparisons between PID088-1 and PID096-A-3 (log₂ odds ratio = 5.41, *p*-value = 0.0001) and between PID088-1 and PID096-A-4 (log₂ odds ratio = 5.64, *p*-value = 3.6 × 10⁻⁶). PID088-1 corresponds to a comparison between lung tumor tissue and normal lung tissue, analyzed by CE-TOFMS. In contrast, PID096-A-3 and PID096-A-4 represent comparisons between tumor and stage-matched normal tissues in stage 0 and stage I colorectal cancer, respectively, which were analyzed using both CE-TOFMS and LC-MS/MS. Despite the differences in organ and cancer type—lung and colorectal cancer—these datasets shared commonly altered metabolites, suggesting the presence of metabolic changes common to early-stage cancer lesions.

A significant positive association was observed between PID047-1 and PID107-1 (log₂ odds ratio = 4.59, *p*-value = 0.008). PID047-1 represents a comparison between MDA-MB-231 breast cancer cells 24 h after arginine deprivation and untreated control cells, measured by LC-LTQ-MS. PID107-1 corresponds to a comparison between anticancer drug-treated and control MSTO-211H malignant pleural mesothelioma cells, analyzed by CE-TOFMS. This result indicates that certain metabolites commonly increased or decreased in response to drug treatment were shared between the two datasets.

A significant positive association was observed between PID018-3 and PID106-D-2 (log₂ odds ratio = 4.76, *p*-value = 0.0101). PID018-3 represents a comparison between MN4-treated and control L428 cells, analyzed by GC-MS and LC-MS. PID106-D-2 corresponds to a comparison between P4HA3-knockdown (shP4HA3) and control (shLacZ) A549 cells (a human lung adenocarcinoma cell line), measured by CE-TOFMS. The presence of commonly altered metabolites suggests that both MN4 treatment and P4HA3 knockdown may affect shared metabolic pathways or molecular networks. P4HA3 has been reported to regulate metabolism and epithelial–mesenchymal transition (EMT) in a TGF-β–dependent manner. MN4 is known to influence cancer metabolism through Sp1-mediated transcriptional regulation [87]. Sp1 has been identified as a transcriptional regulator of P4HA2 in colorectal cancer [88], and in lung and pancreatic adenocarcinomas, it has been reported to suppress EMT via the regulation of LOXL2 [89], [90]. These findings suggest that MN4-mediated suppression of Sp1 may affect EMT-related metabolic and transcriptional networks, potentially leading to metabolic changes similar to those observed in P4HA3 knockdown. Therefore, despite differences in cell type and treatment conditions, the significant association between PID018-3 and PID106-D-2 may reflect their common involvement in EMT suppression.

A significant negative association was observed between PID081-C-1 and PID099-A-1 (log₂ odds ratio = −5.89, *p*-value = 2.53 × 10⁻⁴). PID081-C-1 represents a comparison between tumor and non-tumor tissues in SCLC, analyzed by CE-TOFMS. PID099-A-1 corresponds to a comparison between LLGL2-knockdown and shGFP control breast cancer cells, analyzed by LC-MS/MS. The negative log₂ odds ratio indicates that metabolites increased in LLGL2-knockdown breast cancer cells overlapped with those decreased in SCLC tumor tissues.

A significant negative association was observed between PID017-1 and PID081-A-1 (log₂ odds ratio = −5.36, *p*-value = 0.004). PID017-1 represents a comparison between tumor and normal tissues in ccRCC, analyzed by GC-MS and LC-MS/MS. PID081-A-1 corresponds to a comparison between gedatolisib-treated and DMSO-treated control H446 SCLC, measured by CE-TOFMS. The negative log₂ odds ratio indicates that metabolites increased by gedatolisib treatment overlapped with those decreased in ccRCC tumor tissues. The PI3K/mTOR pathway is known to be activated in various cancer types and plays a key role in metabolic reprogramming. Inhibition of this pathway by gedatolisib may suppress or normalize cancer-specific metabolic alterations. Therefore, the observed negative association may reflect a partial restoration of tumor-associated metabolism in response to therapeutic intervention.

A significant negative association was observed between PID092-4 [65] and PID098-B-1 [71] (log₂ odds ratio = −4.27, *p*-value = 0.0074). PID092-4 represents a comparison between liver-specific Atg7-knockout mutant mice and control mice, analyzed by CE-TOFMS. PID098-B-1 corresponds to a comparison between PKM1-expressing (PkmM1) and PKM2-expressing (PkmM2) mouse embryonic fibroblasts treated with BEZ235 for 2 days, measured by CE-TOFMS and LC-MS/MS. Commonly altered metabolites included glycolysis-related compounds such as 3-phosphoglycerate, D-fructose 6-phosphate, and glucose 6-phosphate, as well as redox-related metabolites including ophthalmate and cysteine-glutathione disulfide. Previous studies have shown that PKM1-expressing cells exhibit higher autophagic activity than PKM2-expressing cells, and that PKM1 may drive elevated glucose metabolism and autophagy in tumor cells [71]. The present comparison highlights shared metabolic changes between two mouse models with contrasting autophagy states—autophagy-deficient mice (PID092-4) and PKM1-driven metabolic cells (PID098-B-1). These findings may offer new insights into the relationship between autophagy and tumor metabolism. Further elucidation of the mechanism by which PKM1 promotes autophagy may enhance our understanding of metabolic flexibility and adaptive strategies in cancer.

**References**

[1] D. Monleón *et al.*, “Metabolic aggressiveness in benign meningiomas with chromosomal instabilities,” *Cancer Res*, vol. 70, no. 21, 2010, doi: 10.1158/0008-5472.CAN-10-1498.

[2] B. Roe, E. Kensicki, R. Mohney, and W. W. Hall, “Metabolomic profile of hepatitis C Virus-Infected hepatocytes,” *PLoS One*, vol. 6, no. 8, 2011, doi: 10.1371/journal.pone.0023641.

[3] C. Frezza *et al.*, “Metabolic profiling of hypoxic cells revealed a catabolic signature required for cell survival,” *PLoS One*, vol. 6, no. 9, 2011, doi: 10.1371/journal.pone.0024411.

[4] T. Yoshie *et al.*, “Regulation of the metabolite profile by an APC gene mutation in colorectal cancer,” *Cancer Sci*, vol. 103, no. 6, 2012, doi: 10.1111/j.1349-7006.2012.02262.x.

[5] C. Quijano *et al.*, “Oncogene-induced senescence results in marked metabolic and bioenergetic alterations,” *Cell Cycle*, vol. 11, no. 7, 2012, doi: 10.4161/cc.19800.

[6] H. L. Kotze *et al.*, “A novel untargeted metabolomics correlation-based network analysis incorporating human metabolic reconstructions,” *BMC Syst Biol*, vol. 7, 2013, doi: 10.1186/1752-0509-7-107.

[7] L. Brunelli, E. Caiola, M. Marabese, M. Broggini, and R. Pastorelli, “Capturing the metabolomic diversity of KRAS mutants in non-small-cell lung cancer cells,” *Oncotarget*, vol. 5, no. 13, 2014, doi: 10.18632/oncotarget.1958.

[8] L. M. Poisson *et al.*, “A metabolomic approach to identifying platinum resistance in ovarian cancer,” *J Ovarian Res*, vol. 8, no. 1, 2015, doi: 10.1186/s13048-015-0140-8.

[9] T. W. Miller *et al.*, “CD47 receptor globally regulates metabolic pathways that control resistance to ionizing radiation,” *Journal of Biological Chemistry*, vol. 290, no. 41, 2015, doi: 10.1074/jbc.M115.665752.

[10] Y. Amano *et al.*, “Metabolic alterations caused by HNF1ß expression in ovarian clear cell carcinoma contribute to cell survival,” *Oncotarget*, vol. 6, no. 28, 2015, doi: 10.18632/oncotarget.4692.

[11] A. Wojakowska *et al.*, “Detection of metabolites discriminating subtypes of thyroid cancer: Molecular profiling of FFPE samples using the GC/MS approach,” *Mol Cell Endocrinol*, vol. 417, 2015, doi: 10.1016/j.mce.2015.09.021.

[12] S. Ganti *et al.*, “Kidney tumor biomarkers revealed by simultaneous multiple matrix metabolomics analysis,” *Cancer Res*, vol. 72, no. 14, 2012, doi: 10.1158/0008-5472.CAN-11-3105.

[13] H. Makinoshima *et al.*, “Signaling through the phosphatidylinositol 3-kinase (PI3K)/mammalian target of rapamycin (mTOR) axis is responsible for aerobic glycolysis mediated by glucose transporter in epidermal growth factor receptor (EGFR)-mutated lung adenocarcinoma,” *Journal of Biological Chemistry*, vol. 290, no. 28, 2015, doi: 10.1074/jbc.M115.660498.

[14] E. G. Armitage, H. L. Kotze, J. W. Allwood, W. B. Dunn, R. Goodacre, and K. J. Williams, “Metabolic profiling reveals potential metabolic markers associated with Hypoxia Inducible Factor-mediated signalling in hypoxic cancer cells,” *Sci Rep*, vol. 5, 2015, doi: 10.1038/srep15649.

[15] S. Meller *et al.*, “Integration of tissue metabolomics, transcriptomics and immunohistochemistry reveals ERG- and gleason score- specific metabolomic alterations in prostate cancer,” *Oncotarget*, vol. 7, no. 2, 2016, doi: 10.18632/oncotarget.6370.

[16] Salony *et al.*, “AKT inhibition promotes nonautonomous cancer cell survival,” *Mol Cancer Ther*, vol. 15, no. 1, 2016, doi: 10.1158/1535-7163.MCT-15-0414.

[17] A. A. Hakimi *et al.*, “An Integrated Metabolic Atlas of Clear Cell Renal Cell Carcinoma,” *Cancer Cell*, vol. 29, no. 1, 2016, doi: 10.1016/j.ccell.2015.12.004.

[18] K. Kimura and R. C. C. Huang, “Tetra-O-Methyl nordihydroguaiaretic acid broadly suppresses cancer metabolism and synergistically induces strong anticancer activity in combination with etoposide, rapamycin and UCN-01,” *PLoS One*, vol. 11, no. 2, 2016, doi: 10.1371/journal.pone.0148685.

[19] K. Fujisawa *et al.*, “Modulation of anti-cancer drug sensitivity through the regulation of mitochondrial activity by adenylate kinase 4,” *Journal of Experimental and Clinical Cancer Research*, vol. 35, no. 1, 2016, doi: 10.1186/s13046-016-0322-2.

[20] G. Deep *et al.*, “Silibinin inhibits hypoxia-induced HIF-1α-mediated signaling, angiogenesis and lipogenesis in prostate cancer cells: In vitro evidence and in vivo functional imaging and metabolomics,” *Mol Carcinog*, vol. 56, no. 3, 2017, doi: 10.1002/mc.22537.

[21] L. Tang *et al.*, “Global metabolic profiling identifies a pivotal role of proline and hydroxyproline metabolism in supporting hypoxic response in hepatocellular carcinoma,” *Clinical Cancer Research*, vol. 24, no. 2, 2018, doi: 10.1158/1078-0432.CCR-17-1707.

[22] Y. K. Al-Mutawa, A. Herrmann, C. Corbishley, P. D. Losty, M. Phelan, and V. Sée, “Effects of hypoxic preconditioning on neuroblastoma tumour oxygenation and metabolic signature in a chick embryo model,” *Biosci Rep*, vol. 38, no. 4, 2018, doi: 10.1042/BSR20180185.

[23] J. M. Ayuso *et al.*, “Organotypic microfluidic breast cancer model reveals starvation-induced spatial-temporal metabolic adaptations,” *EBioMedicine*, vol. 37, 2018, doi: 10.1016/j.ebiom.2018.10.046.

[24] G. Lucarelli *et al.*, “Integrated multi-omics characterization reveals a distinctive metabolic signature and the role of NDUFA4L2 in promoting angiogenesis, chemoresistance, and mitochondrial dysfunction in clear cell renal cell carcinoma,” *Aging*, vol. 10, no. 12, 2018, doi: 10.18632/aging.101685.

[25] J. Yang *et al.*, “Untargeted and stable isotope-assisted metabolomic analysis of MDA-MB-231 cells under hypoxia,” *Metabolomics*, vol. 14, no. 4, 2018, doi: 10.1007/s11306-018-1338-8.

[26] W. D. Lee, D. Mukha, E. Aizenshtein, and T. Shlomi, “Spatial-fluxomics provides a subcellular-compartmentalized view of reductive glutamine metabolism in cancer cells,” *Nat Commun*, vol. 10, no. 1, 2019, doi: 10.1038/s41467-019-09352-1.

[27] H. Li *et al.*, “The landscape of cancer cell line metabolism,” *Nat Med*, vol. 25, no. 5, 2019, doi: 10.1038/s41591-019-0404-8.

[28] S. Ishikawa *et al.*, “Identification of salivary metabolomic biomarkers for oral cancer screening,” *Sci Rep*, vol. 6, 2016, doi: 10.1038/srep31520.

[29] T. Itoi *et al.*, “Serum metabolomic profiles for human pancreatic cancer discrimination,” *Int J Mol Sci*, vol. 18, no. 4, 2017, doi: 10.3390/ijms18040767.

[30] S. Yachida *et al.*, “Metagenomic and metabolomic analyses reveal distinct stage-specific phenotypes of the gut microbiota in colorectal cancer,” *Nat Med*, vol. 25, no. 6, 2019, doi: 10.1038/s41591-019-0458-7.

[31] P. P. Erawijantari *et al.*, “Influence of gastrectomy for gastric cancer treatment on faecal microbiome and metabolome profiles,” *Gut*, vol. 69, no. 8, 2020, doi: 10.1136/gutjnl-2019-319188.

[32] Y. Sato *et al.*, “Metabolic characterization of antifolate responsiveness and nonresponsiveness in malignant pleural mesothelioma cells,” *Front Pharmacol*, vol. 9, no. OCT, 2018, doi: 10.3389/fphar.2018.01129.

[33] A. Maruyama *et al.*, “De novo deoxyribonucleotide biosynthesis regulates cell growth and tumor progression in small-cell lung carcinoma,” *Sci Rep*, vol. 11, no. 1, 2021, doi: 10.1038/s41598-021-92948-9.

[34] H. Primasová *et al.*, “1H HR-MAS NMR-based metabolomics of cancer cells in response to treatment with the diruthenium trithiolato complex [(P-MeC6H4iPr)2Ru2(SC6H4-p-But)3]+ (DiRu-1),” *Metabolites*, vol. 9, no. 7, 2019, doi: 10.3390/metabo9070146.

[35] D. Licha *et al.*, “Untargeted metabolomics reveals molecular effects of ketogenic diet on healthy and tumor xenograft mouse models,” *Int J Mol Sci*, vol. 20, no. 16, 2019, doi: 10.3390/ijms20163873.

[36] Y. Cai *et al.*, “Sex Differences in Colon Cancer Metabolism Reveal A Novel Subphenotype,” *Sci Rep*, vol. 10, no. 1, 2020, doi: 10.1038/s41598-020-61851-0.

[37] X. Shen *et al.*, “Asparagine Metabolism in Tumors Is Linked to Poor Survival in Females with Colorectal Cancer: A Cohort Study,” *Metabolites*, vol. 12, no. 2, 2022, doi: 10.3390/metabo12020164.

[38] D. P. Labbé *et al.*, “High-fat diet fuels prostate cancer progression by rewiring the metabolome and amplifying the MYC program,” *Nat Commun*, vol. 10, no. 1, 2019, doi: 10.1038/s41467-019-12298-z.

[39] H. W. Ressom *et al.*, “Utilization of metabolomics to identify serum biomarkers for hepatocellular carcinoma in patients with liver cirrhosis,” *Anal Chim Acta*, vol. 743, 2012, doi: 10.1016/j.aca.2012.07.013.

[40] V. J. Bhute and S. P. Palecek, “Metabolic responses induced by DNA damage and poly (ADP-ribose) polymerase (PARP) inhibition in MCF-7 cells,” *Metabolomics*, vol. 11, no. 6, 2015, doi: 10.1007/s11306-015-0831-6.

[41] S. Dhakshinamoorthy, N. T. Dinh, J. Skolnick, and M. P. Styczynski, “Metabolomics identifies the intersection of phosphoethanolamine with menaquinone-triggered apoptosis in an in vitro model of leukemia,” *Mol Biosyst*, vol. 11, no. 9, 2015, doi: 10.1039/c5mb00237k.

[42] A. Singh *et al.*, “1H NMR metabolomics reveals association of high expression of inositol 1, 4, 5 trisphosphate receptor and metabolites in breast cancer patients,” *PLoS One*, vol. 12, no. 1, 2017, doi: 10.1371/journal.pone.0169330.

[43] C. D. Hart *et al.*, “Serum metabolomic profiles identify ER-positive early breast cancer patients at increased risk of disease recurrence in a multicenter population,” *Clinical Cancer Research*, vol. 23, no. 6, 2017, doi: 10.1158/1078-0432.CCR-16-1153.

[44] T. H. More *et al.*, “Metabolomic alterations in invasive ductal carcinoma of breast: A comprehensive metabolomic study using tissue and serum samples,” *Oncotarget*, vol. 9, no. 2, 2018, doi: 10.18632/oncotarget.23626.

[45] E. Abad *et al.*, “Common metabolic pathways implicated in resistance to chemotherapy point to a key mitochondrial role in breast cancer,” *Molecular and Cellular Proteomics*, vol. 18, no. 2, 2019, doi: 10.1074/mcp.RA118.001102.

[46] M. E. Knott, M. Manzi, N. Zabalegui, M. O. Salazar, L. I. Puricelli, and M. E. Monge, “Metabolic Footprinting of a Clear Cell Renal Cell Carcinoma in Vitro Model for Human Kidney Cancer Detection,” *J Proteome Res*, vol. 17, no. 11, 2018, doi: 10.1021/acs.jproteome.8b00538.

[47] C. T. Cheng *et al.*, “Arginine starvation kills tumor cells through aspartate exhaustion and mitochondrial dysfunction,” *Commun Biol*, vol. 1, no. 1, 2018, doi: 10.1038/s42003-018-0178-4.

[48] R. Taware *et al.*, “Volatilomic insight of head and neck cancer via the effects observed on saliva metabolites,” *Sci Rep*, vol. 8, no. 1, 2018, doi: 10.1038/s41598-018-35854-x.

[49] H. A. Brauer *et al.*, “Impact of tumor microenvironment and epithelial phenotypes on metabolism in breast cancer,” *Clinical Cancer Research*, vol. 19, no. 3, 2013, doi: 10.1158/1078-0432.CCR-12-2123.

[50] J. Zhu *et al.*, “Colorectal cancer detection using targeted serum metabolic profiling,” *J Proteome Res*, vol. 13, no. 9, 2014, doi: 10.1021/pr500494u.

[51] N. Kumar, M. A. Hoque, and M. Sugimoto, “Robust volcano plot: Identification of differential metabolites in the presence of outliers,” *BMC Bioinformatics*, vol. 19, no. 1, 2018, doi: 10.1186/s12859-018-2117-2.

[52] J. F. Fahrmann *et al.*, “Serum phosphatidylethanolamine levels distinguish benign from malignant solitary pulmonary nodules and represent a potential diagnostic biomarker for lung cancer,” *Cancer Biomarkers*, vol. 16, no. 4, 2016, doi: 10.3233/CBM-160602.

[53] S. Miyamoto *et al.*, “Systemic metabolomic changes in blood samples of lung cancer patients identified by gas chromatography Time-Of-Flight mass spectrometry,” *Metabolites*, vol. 5, no. 2, 2015, doi: 10.3390/metabo5020192.

[54] A. Batova *et al.*, “Englerin A induces an acute inflammatory response and reveals lipid metabolism and ER stress as targetable vulnerabilities in renal cell carcinoma,” *PLoS One*, vol. 12, no. 3, 2017, doi: 10.1371/journal.pone.0172632.

[55] M. N. Aslam *et al.*, “A calcium-rich multimineral intervention to modulate colonic microbial communities and metabolomic profiles in humans: Results from a 90-day trial,” *Cancer Prevention Research*, vol. 13, no. 1, 2020, doi: 10.1158/1940-6207.CAPR-19-0325.

[56] B. Bishal Paudel *et al.*, “An integrative gene expression and mathematical flux balance analysis identifies targetable redox vulnerabilities in melanoma cells,” *Cancer Res*, vol. 80, no. 20, 2021, doi: 10.1158/0008-5472.CAN-19-3588.

[57] E. L. Giddings *et al.*, “Mitochondrial ATP fuels ABC transporter-mediated drug efflux in cancer chemoresistance,” *Nat Commun*, vol. 12, no. 1, 2021, doi: 10.1038/s41467-021-23071-6.

[58] H. Makinoshima *et al.*, “Epidermal growth factor receptor (EGFR) signaling regulates global metabolic pathways in EGFR-mutated lung adenocarcinoma,” *Journal of Biological Chemistry*, vol. 289, no. 30, 2014, doi: 10.1074/jbc.M114.575464.

[59] H. Makinoshima *et al.*, “Metabolic determinants of sensitivity to phosphatidylinositol 3-kinase pathway inhibitor in small-cell lung carcinoma,” *Cancer Res*, vol. 78, no. 9, 2018, doi: 10.1158/0008-5472.CAN-17-2109.

[60] A. Hirayama *et al.*, “Quantitative metabolome profiling of colon and stomach cancer microenvironment by capillary electrophoresis time-of-flight mass spectrometry,” *Cancer Res*, vol. 69, no. 11, 2009, doi: 10.1158/0008-5472.CAN-08-4806.

[61] K. Kami *et al.*, “Metabolomic profiling of lung and prostate tumor tissues by capillary electrophoresis time-of-flight mass spectrometry,” *Metabolomics*, vol. 9, no. 2, 2013, doi: 10.1007/s11306-012-0452-2.

[62] J. Adam *et al.*, “A Role for Cytosolic Fumarate Hydratase in Urea Cycle Metabolism and Renal Neoplasia,” *Cell Rep*, vol. 3, no. 5, 2013, doi: 10.1016/j.celrep.2013.04.006.

[63] D. Kim *et al.*, “SHMT2 drives glioma cell survival in ischaemia but imposes a dependence on glycine clearance,” *Nature*, vol. 520, no. 7547, 2015, doi: 10.1038/nature14363.

[64] J. L. Coloff *et al.*, “Differential Glutamate Metabolism in Proliferating and Quiescent Mammary Epithelial Cells,” *Cell Metab*, vol. 23, no. 5, 2016, doi: 10.1016/j.cmet.2016.03.016.

[65] T. Saito *et al.*, “P62/Sqstm1 promotes malignancy of HCV-positive hepatocellular carcinoma through Nrf2-dependent metabolic reprogramming,” *Nat Commun*, vol. 7, 2016, doi: 10.1038/ncomms12030.

[66] D. Karigane *et al.*, “p38α Activates Purine Metabolism to Initiate Hematopoietic Stem/Progenitor Cell Cycling in Response to Stress,” *Cell Stem Cell*, vol. 19, no. 2, 2016, doi: 10.1016/j.stem.2016.05.013.

[67] J. C. Kremer *et al.*, “Arginine Deprivation Inhibits the Warburg Effect and Upregulates Glutamine Anaplerosis and Serine Biosynthesis in ASS1-Deficient Cancers,” *Cell Rep*, vol. 18, no. 4, 2017, doi: 10.1016/j.celrep.2016.12.077.

[68] S. Tabata *et al.*, “Thymidine Catabolism as a Metabolic Strategy for Cancer Survival,” *Cell Rep*, vol. 19, no. 7, 2017, doi: 10.1016/j.celrep.2017.04.061.

[69] K. Satoh *et al.*, “Global metabolic reprogramming of colorectal cancer occurs at adenoma stage and is induced by MYC,” *Proc Natl Acad Sci U S A*, vol. 114, no. 37, 2017, doi: 10.1073/pnas.1710366114.

[70] H. Sakagami *et al.*, “Effects of 3-styrylchromones on metabolic profiles and cell death in oral squamous cell carcinoma cells,” *Toxicol Rep*, vol. 2, 2015, doi: 10.1016/j.toxrep.2015.09.009.

[71] M. Morita *et al.*, “PKM1 Confers Metabolic Advantages and Promotes Cell-Autonomous Tumor Cell Growth,” *Cancer Cell*, vol. 33, no. 3, 2018, doi: 10.1016/j.ccell.2018.02.004.

[72] Y. Saito *et al.*, “LLGL2 rescues nutrient stress by promoting leucine uptake in ER+ breast cancer,” *Nature*, vol. 569, no. 7755, 2019, doi: 10.1038/s41586-019-1126-2.

[73] Y. Yang *et al.*, “Integrated microbiome and metabolome analysis reveals a novel interplay between commensal bacteria and metabolites in colorectal cancer,” *Theranostics*, vol. 9, no. 14, 2019, doi: 10.7150/thno.35186.

[74] T. Osawa *et al.*, “Phosphoethanolamine Accumulation Protects Cancer Cells under Glutamine Starvation through Downregulation of PCYT2,” *Cell Rep*, vol. 29, no. 1, 2019, doi: 10.1016/j.celrep.2019.08.087.

[75] N. Harada-Shoji *et al.*, “A metabolic profile of routine needle biopsies identified tumor type specific metabolic signatures for breast cancer stratification: a pilot study,” *Metabolomics*, vol. 15, no. 11, 2019, doi: 10.1007/s11306-019-1610-6.

[76] N. Kikuchi *et al.*, “Comparison of the ischemic and non-ischemic lung cancer metabolome reveals hyper activity of the TCA cycle and autophagy,” *Biochem Biophys Res Commun*, vol. 530, no. 1, 2020, doi: 10.1016/j.bbrc.2020.07.082.

[77] M. Sato *et al.*, “L-type amino acid transporter 1 is associated with chemoresistance in breast cancer via the promotion of amino acid metabolism,” *Sci Rep*, vol. 11, no. 1, 2021, doi: 10.1038/s41598-020-80668-5.

[78] J. Guo, K. Satoh, S. Tabata, M. Mori, M. Tomita, and T. Soga, “Reprogramming of glutamine metabolism via glutamine synthetase silencing induces cisplatin resistance in A2780 ovarian cancer cells,” *BMC Cancer*, vol. 21, no. 1, 2021, doi: 10.1186/s12885-021-07879-5.

[79] F. Nakasuka *et al.*, “TGF-β-dependent reprogramming of amino acid metabolism induces epithelial–mesenchymal transition in non-small cell lung cancers,” *Commun Biol*, vol. 4, no. 1, 2021, doi: 10.1038/s42003-021-02323-7.

[80] Y. Sato, M. Tomita, T. Soga, A. Ochiai, and H. Makinoshima, “Upregulation of Thymidylate Synthase Induces Pemetrexed Resistance in Malignant Pleural Mesothelioma,” *Front Pharmacol*, vol. 12, 2021, doi: 10.3389/fphar.2021.718675.

[81] Y. Saito *et al.*, “Polarity protein SCRIB interacts with SLC3A2 to regulate proliferation and tamoxifen resistance in ER+ breast cancer,” *Commun Biol*, vol. 5, no. 1, 2022, doi: 10.1038/s42003-022-03363-3.

[82] T. Hoshii *et al.*, “SETD1A regulates transcriptional pause release of heme biosynthesis genes in leukemia,” *Cell Rep*, vol. 41, no. 9, 2022, doi: 10.1016/j.celrep.2022.111727.

[83] Y. Murakami *et al.*, “Comprehensive analysis of transcriptome and metabolome analysis in Intrahepatic Cholangiocarcinoma and Hepatocellular Carcinoma,” *Sci Rep*, vol. 5, 2015, doi: 10.1038/srep16294.

[84] L. V. Albrecht *et al.*, “GSK3 Inhibits Macropinocytosis and Lysosomal Activity through the Wnt Destruction Complex Machinery,” *Cell Rep*, vol. 32, no. 4, 2020, doi: 10.1016/j.celrep.2020.107973.

[85] P. Shriwas *et al.*, “A small-molecule pan-class I glucose transporter inhibitor reduces cancer cell proliferation in vitro and tumor growth in vivo by targeting glucose-based metabolism,” *Cancer Metab*, vol. 9, no. 1, 2021, doi: 10.1186/s40170-021-00248-7.

[86] C. Caslini, S. Hong, Y. J. Ban, X. S. Chen, and T. A. Ince, “HDAC7 regulates histone 3 lysine 27 acetylation and transcriptional activity at super-enhancer-associated genes in breast cancer stem cells,” *Oncogene*, vol. 38, no. 39, 2019, doi: 10.1038/s41388-019-0897-0.

[87] R. Bajpai and G. P. Nagaraju, “Specificity protein 1: Its role in colorectal cancer progression and metastasis,” 2017. doi: 10.1016/j.critrevonc.2017.02.024.

[88] X. Dang *et al.*, “P4HA2 promotes tumor progression and is transcriptionally regulated by SP1 in colorectal cancer,” *Cancer Biol Ther*, vol. 25, no. 1, 2024, doi: 10.1080/15384047.2024.2361594.

[89] L. Xu, W. L. Liao, Q. J. Lu, P. Zhang, J. Zhu, and G. N. Jiang, “Hypoxic tumor-derived exosomal circular RNA SETDB1 promotes invasive growth and EMT via the miR-7/Sp1 axis in lung adenocarcinoma,” 2021. doi: 10.1016/j.omtn.2021.01.019.

[90] N. Kolesnikoff *et al.*, “Specificity protein 1 (Sp1) maintains basal epithelial expression of the mir-200 family: Implications for epithelial-mesenchymal transition,” *Journal of Biological Chemistry*, vol. 289, no. 16, 2014, doi: 10.1074/jbc.M113.529172.

**Supplementary Figure 1. Overview of the analytical workflow.**

This figure illustrates the
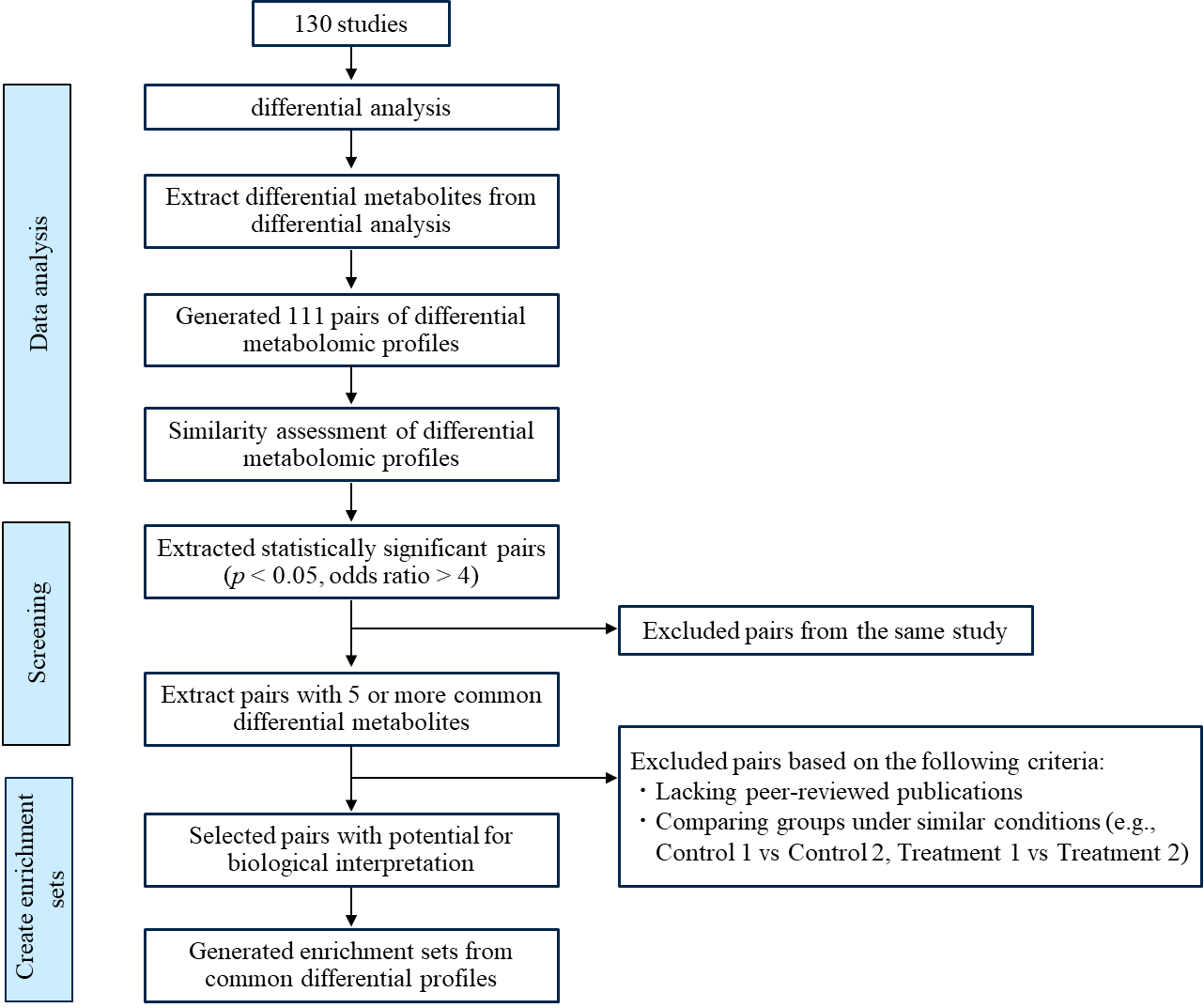
 overall workflow of the analysis. It outlines the process of identifying commonly varying metabolite sets using the iDMET approach and integrating them into enrichment analysis.
